# Molecular Cloning and Functional Analysis of Squalene Synthase 2(SQS2) in *Salvia miltiorrhiza* Bunge

**DOI:** 10.3389/fpls.2016.01274

**Published:** 2016-08-24

**Authors:** Qixian Rong, Dan Jiang, Yijun Chen, Ye Shen, Qingjun Yuan, Huixin Lin, Liangping Zha, Yan Zhang, Luqi Huang

**Affiliations:** ^1^State Key Laboratory Breeding Base of Dao-di Herbs, National Resource Center for Chinese Materia Medica, China Academy of Chinese Medical SciencesBeijing, China; ^2^School of Chinese Pharmacy, Beijing University of Chinese MedicineBeijing, China; ^3^Jiangxi University of Traditional Chinese MedicineNanchang, China

**Keywords:** *S. miltiorrhiza* Bunge, squalene synthase 2, functional characterization, GC–MS, qRT-PCR

## Abstract

*Salvia miltiorrhiza* Bunge, which is also known as a traditional Chinese herbal medicine, is widely studied for its ability to accumulate the diterpene quinone Tanshinones. In addition to producing a variety of diterpene quinone, *S. miltiorrhiza* Bunge also accumulates sterol, brassinosteroid and triterpenoids. During their biosynthesis, squalene synthase (SQS, EC 2.5.1.21) converts two molecules of the hydrophilic substrate farnesyl diphosphate (FPP) into a hydrophobic product, squalene. In the present study, cloning and characterization of *S. miltiorrhiza* Bunge squalene synthase 2 (SmSQS2, Genbank Accession Number: KM408605) cDNA was investigated subsequently followed by its recombinant expression and preliminary enzyme activity. The full-length cDNA of *SmSQS2* was 1 597 bp in length, with an open reading frame of 1 245 bp encoding 414 amino acids. The deduced amino acid sequence of SmSQS2 shared high similarity with those of SQSs from other plants. To obtain soluble recombinant enzymes, the truncated SmSQS2 in which 28 amino acids were deleted from the carboxy terminus was expressed as GST-Tag fusion protein in *Escherichia coli* BL21 (DE3) and confirmed by SDS-PAGE and Western Blot analysis, and the resultant bacterial crude extract was incubated with FPP and NADPH. Gas chromatograph-mass spectrometer analysis showed that squalene was detected in the *in vitro* reaction mixture. The gene expression level was analyzed through Quantitative real-time PCR, and was found to be higher in roots as compared to the leaves, and was up-regulated upon YE+ Ag^+^ treatment. These results could serve as an important to understand the function of the *SQS* family. In addition, the identification of SmSQS2 is important for further studies of terpenoid and sterol biosynthesis in *S. miltiorrhiza* Bunge.

## Introduction

Sterols (also called phytosterols) and triterpenoid are widely distributed isoprenoids, and they constitute one of the most important classes of natural products. In plants, the dominant sterols are 24-alkyl sterols including campesterol, stigmasterol, and sitosterol while the other non-methylated C-24 sterols, such as cholesterol, are present in relatively low amounts (Gunstone et al., 2007). Most plant triterpenoid compounds are in the form of saponin glycosides, which refers to the attachment of various sugar molecules to the triterpene unit. Triterpenes are ubiquitous isoprenoids produced by all eukaryotes and have various applications as drugs, cosmetics and other commercial applications (Hostettmann and Marston, 1995).

Both plant sterols and triterpenoid saponins are synthesized from the same precursors, squalene (Haralampidis et al., 2002). Squalene has been extensively investigated by the medical and pharmaceutical sectors because many studies have shown that the chemical effectively inhibits chemically induced colon, lung, and skin cancers; has bactericidal and antifungal properties; markedly increases both cellular and non-specific immune functions; and reduces serum cholesterol levels (Kim and Karadeniz, 2012).

Squalene synthase (SQS, E.C. 2.5.1.21) is a membrane-bound enzyme catalyzing the first dedicated step in the biosynthesis of sterols and other triterpenoids (Abe et al., 1993), and it is a bifunctional enzyme initially catalyzing the condensation of two molecules of farnesyl diphosphate (FPP) to form presqualene diphosphate (PSPP), and next converting PSPP to squalene (SQ) in a reaction requiring NADPH and Mg^2+^ (Takatsuji et al., 1982; Zhao et al., 2010).

As SQS is a key enzyme regulating isoprenoid biosynthesis, the genes encoding the enzyme have been cloned from protozoa (Okada et al., 2000), bacteria (Lee and Poulter, 2008), fungi (LoGrasso et al., 1993; Zhang et al., 1993), plants (Devarenne et al., 1998; Huang et al., 2007; Busquets et al., 2008), animals (McKenzie et al., 1992), and human beings (Summers et al., 1993). Almost all studies of SQSs have attracted great attention their role in the biosynthesis of sterols and triterpenoid.

In plants, like many other enzymes of the MVA pathway, is encoded by small gene families. A single SQS gene has been reported in many plants, such as *Taxus cuspidata* (Huang et al., 2007), *Euphorbia tirucalli* (Uchida et al., 2009), *Oryza sativa* (Hata et al., 1997), *Lotus japonicus* (Akamine et al., 2003), *Chlorophytum borivilianum* (Kalra et al., 2013), and *E. pekinensis* (Zheng et al., 2013), while it has been found that there are two SQS genes in *Arabidopsis thaliana* (Kribii et al., 1997), tobacco (Devarenne et al., 1998), *Glycyrrhiza glabra* (Hayashi et al., 1999), *Withania somnifera* (Gupta et al., 2012), *Glycine max* (Nguyen et al., 2013), and *Salvia miltiorrhiza* Bunge (Ma et al., 2014), and three SQS genes in *Panax ginseng* (Kim et al., 2011).

The genome of *A. thaliana* contains two SQS-annotated sequences, At4g34640 (*SQS1*) and At4g34650 (*SQS2*), organized in tandem array (Kribii et al., 1997). The *A. thaliana* SQS1 gene is widely expressed in all tissues throughout plant development, whereas SQS2 is primarily expressed in the vascular tissue of leaf and cotyledon petioles, and the hypocotyl of seedlings (Busquets et al., 2008). The recombinant SQS2 was unable to synthesize SQ from FPP in the presence of NADPH and either Mg^2+^ or Mn^2+^, whereas under the same assay conditions an equivalent preparation of SQS1 efficiently transformed FPP into SQ, so that SQS1 is the only functional SQS in *A. thaliana* (Busquets et al., 2008). However, *SQS1* and *SQS2* from *G. max* were able to convert yeast sterol auxotrophy erg9 mutant to sterol prototrophy and overexpression of *GmSQS1* increased end product sterols in *Arabidopsis* seeds (Nguyen et al., 2013). In addition, all three *SQS* genes (*SS1*, *SS2*, and *SS3*) were identified in *P. ginseng* were able to convert yeast erg9 mutant cells to ergosterol prototrophy in spite of sequence divergence to yeast (Kim et al., 2011). Likewise, similar results have been reported in *W. somnifera* squalene synthase (*WsSQS1*and*WsSQS2*) cDNA was investigated subsequently followed by its recombinant expression and preliminary enzyme activity (Gupta et al., 2012).

*Salvia miltiorrhizais* Bunge, also known as “Danshen,” is an important traditional Chinese medicine that has recorded medical usage dating back to nearly 2,000 years ago. Danshen mainly used in the clinical treatment of cardiovascular and cerebrovascular diseases in China, Japan, and other Asian countries (Zhou et al., 2005). The main effective elements of Radix *S. miltiorrhizais* Bunge are Tanshinones, which are abietane-type norditerpenoid quinones and has strong physiological activities (Wang et al., 2007; Dong et al., 2011; Yang et al., 2013). In addition, the bioactive compounds of *S. miltiorrhizais* Bunge including sterols, which capacity to modulate the ordering of lipids is critical for membrane organization (Goldstein et al., 2006), and triterpenoid with a wide range of structural diversity and biological activity (Hostettmann and Marston, 1995), which synthesized from the same precursors, squalene and 2,3-oxidosqualene (Haralampidis et al., 2002). In the previous study two squalene synthase genes, *SmSQS1* and *SmSQS2*, were identified in *S. miltiorrhizais* Bunge (Ma et al., 2014). However, the function of SQS2 is still unexplored in *S. miltiorrhizais* Bunge and whether the SQS2 of *S. miltiorrhizais* Bunge was able to synthesize SQ from FPP in the presence of NADPH and either Mg^2+^ or Mn^2+^. Hence, we describe the cloning, recombinant expression, gene expression, and functional analysis of SQS2 from the *S. miltiorrhizais* Bunge.

## Materials and Methods

### Plant Material

The seeds of *S. miltiorrhiza* Bunge were surface-sterilized with 0.1% HgCl_2_ and cultured on Murashige and Skoog (MS) medium, supplemented with 30 g/L sucrose and 7 g/L agar (PH = 5.6–5.8). Cultures were cultivated in a growth chamber maintained at 25°C, under 16-h-day and 8-h-night regime. After three subcultures, we chose the seedlings that grew well and samples of vegetative tissue (root and leaf) were collected from plants 30 days after subculture. All the samples were frozen directly into liquid nitrogen and stored at -80°C for further analysis.

### Plant Hairy-Root Culture

The *S. miltiorrhiza* hairy-root culture was derived after the infection of plantlets with a Ri (root-inducing) T-DNA (transfer DNA)-bearing *Rhizobium rhizogenes* bacterium (A.T.C.C. 15834; Chen et al., 1999). The *S. miltiorrhiza* hairy root culture used in this work was maintained on hormone-free 6,7-V medium with 30 g/L sucrose at 25°C in the dark. All experiments in this study were carried out in shake-flask cultures with 500-ml Erlenmeyer flasks on an orbital shaker set at 110–120 rpm. Each flask contained 200 ml 6,7-V liquid medium and inoculated with 2 g fresh weight (fw) of roots from 3- to 4-week-old shake-flask cultures.

### Elicitation Experiments

The yeast elicitor (YE) was the carbohydrate fraction of yeast extract prepared by ethanol precipitation (Hahn and Albersheim, 1978). In brief, 25 g of yeast extract was dissolved in 125 mL of distilled water, and then mixed with 100 mL of ethanol. The solution was allowed to precipitate for 4 days at 4°C in a refrigerator, and the supernatant was decanted. The precipitate was redissolved in 125 mL of distilled water and subjected to another round of ethanol precipitation. The final precipitate was dissolved in 100 mL of distilled water, sterilized by autoclaving at 120°C for 20 min and stored at 4°C in a refrigerator before use. Abiotic (Ag^+^) was dissolved in distilled water to the concentration of 3 mM and sterilized by filtration (0.22 μm membrane). Combined elicitors of YE+ Ag^+^ were added to the shake-flask culture of *S. miltiorrhiza* hairy roots on day 18. YE (2 mL) and Ag^+^ (66.7 μL) were added into the culture medium (200 mL). After 0, 1, 2, 4, 8, 12, 24, and 36 h treatment with combined elicitors, hairy roots were collected for SmSQS2 expression analysis. All treatments were performed in triplicate, and the results were averaged.

### Total RNA Isolation and cDNA Synthesis

Total RNA was extracted from roots of *S. miltiorrhiza* Bunge using a TRIzol Reagents (Invitrogen, USA) following the manufacturer’s instructions. RNA quality was verified by an absorbance at 260 nm and 280 nm optical densities using the NanoDrop 2000 Spectrophotometer (Thermo Scientific, San Jose, CA, USA) and by gel electrophoresis (1% agarose). Total RNA was treated using DNase I with an RNAclean kit (BioTeke, Beijing, China), according to the manufacturer’s instructions, to remove any residual DNA. The first-strand cDNA was synthesized using PrimeScript 1st strand cDNA synthesis kit (TaKaRa Biotechnology, Dalian, China). The cDNA was stored at -80°C for further analysis.

### Cloning and Sequencing of *SmSQS2* Full-Length cDNA

All primers used in this study are listed in **Table 1**. The full-length *SmSQS2* cDNA was amplified using SQS2-F and SQS2-R (**Table 1**). The PCR products were purified with a EasyPure Quick Gel Extraction Kit (TransGen Biotech, Beijing, China) and cloned into the pEASY-Blunt Simple Vector (TransGen, Beijing, China). Transformation of *Escherichia coli* DH5α (TransGen, Beijing, China) competent cells was carried out. The recombinant plasmid were isolated and subjected to nucleotide sequencing.

**Table 1:** List of primers sequences used in the current study.

Primer name	Sequence(5′–3′)
SQS2-F	AAAGTCAT CTTTGTATGCCCACA
SQS2-R	TATGTCTGCGTCACCGACTTCCC
SQS2-cF	TTTTTTGTCGACTCATGGTGAATTTGGGGGCGA
SQS2-cR	TTTTTTTTGCGGCCGCTTGTGGCTTCCTCTGGATAATG
SQS2-qF	AGATTGACCTTTATGTTGGC
SQS2-qR	TCAGTGCGTCCGTTGCTTGC
Actin-F	AGGAACCACCGATCCAGACA
Actin-R	GGTGCCCTGAGGTCCTGTT

### Phylogenetic Analysis

The nucleotide and deduced amino acid sequences were analyzed and sequence comparison was conducted through database search using BLAST tool on NCBI^1^. The open reading frame (ORF) was searched using ORF Finder^2^. Putative molecular weight and Theoretical isoelectric point values were calculated using ExPASy tool^3^. Protein Transmembrane Helical Regions was predicted by TRMHMMserver^4^ v2.0. Three dimensional homologous modeling was analyzed by SWISS-MODEL^5^. Multiple sequence alignment was implemented using DNAMAN and ClustalW software. Phylogenetic analysis was constructed using MEGA6 software with the Neighbor-Joining method. Confidence values for individual branches were measured from 1 000 bootstrap replicates of the original sequence data.

### Expression of Recombinant Protein

The coding region for *SmSQS2* without the C-terminal membrane-anchoring signal was amplified using the forward primer SQS2-cF and SQS2-cR (**Table 1**). This PCR product was digested with *Sal* I and *Not* I and cloned into the *Sal* I and *Not* I sites of the expression vector *pGEX-4T-1*. This newly constructed vector, *pGEX-4T-1-SmSQS2*Δ*TM*, was transformed into competent *E. coli* DH5α. Confirmation that the correct construction had been obtained was determined by sequencing. The *pGEX-4T-1-SmSQS2*Δ*TM* was transformed into *E. coli* BL21 (DE3), which was grown at 37°C until the A_600_ of 0.4–0.6 was reached. The expression was induced with 1 mM Isopropylb-D-1-thiogalactopyranoside (IPTG) at 30°C for 6 h. The cells were resuspended in extraction buffer (100 mmol/l Tris–HCl, pH 7.5, 10 mmol/l MgCl_2_, 2% glycerol, and 1 mmol/l DTT) and disrupted by sonication. The lysate was centrifuged at 12,000*g* for 30 min at 4°C, and the supernatant was loaded on 10% SDS-PAGE gel after denaturating with SDS loading dye at 100°C for 5 min.

The gel was stained with coomassie brilliant blue G-250 and decolorized with mixture (60% Ultrapure water; 30% anhydrous alcohol; 10% acetic acid; Kalra et al., 2013).

### Western Blotting

Recombinant protein were analyzed by 10% SDS-PAGE gel and transferred onto polyvinylidene fluoride (PVDF) membrane. The membrane was washed with confining liquid (PBS and blocked with 5% non-fat dry milk) for 1 h with 50 rpm in room temperature (RT). Then the membrane was incubated with mouse polyclonal antibodies against GST (1:10,000, EASYBIO., Beijin, China) at 4°C for overnight. After that, the membrane was washed four times with 1x PBST buffer (10 mM Tris-HCl, pH 8.0, 150 mM NaCl and 0.05% Tween 20) for 5 min. After a final wash with 1x PBST buffer, the membrane was incubated for 1 h with the antibody goat anti-mouse IgG (H&L)-HRP conjugated (1:5,000). After that, the membrane was washed three times with PBST buffer (5 min each). The membrane was then visualized using the Super Enhanced chemilumin escence detection kit (Applygen Technologies Inc., Beijing, China) and Fluorescence was detected using Kodak X-OMAT film (CARESTREAM, Xiamen, China). The films were photographed under UMAX PowerLook 2100XL-USB.

### Enzyme Activity of SQS2

The activity of squalene synthase formation was assayed as follows (Ye et al., 2014). The 500 μL reaction mixture contained 100 mM Tris–HCl (pH 7.5), 10 mM farnesyl pyrophosphate triammonium salt (FPP; Sigma–Aldrich, USA), 10 mM MgCl_2_, 1 mM DTT, 2% Glycine, 3 mM NADPHNa_4_ and 475 μL crude enzyme solution prepared from the *E. coli* cells. After incubation at 32°C for 10 h, the reaction mixture was extracted three times with 500 μL hexane and three parallel experiments were reacted. This was concentrated using N_2_ bubbles and then the concentrated organic phase was subjected to a gas chromatograph-mass spectrometer (GC-MS) for squalene detection.

The GC-MS analysis was performed on a Trace 1310 (Thermo Scientific, San Jose, CA, USA) coupled with a TSQ 8000 mass selective detector (Thermo Scientific, San Jose, CA, USA). Separation of the analyses was performed using a TG-5MS column (30 m × 0.25 mm I.D. × 0.25 μm film thickness; Thermo Scientific, USA) and helium was used as a carrier gas at a constant flow rate of 1.0 mL/min. The column temperature was maintained at 120°C for 3 min, elevated to 180°C at 15°C/min and then elevated to 260°C at 25°C/min. The injection volume was 1 μL for authentic squalene (National Institute for the Control of Pharmaceutical and Biological Products, China) and samples. Mass spectra, 70 eV (in EI mode), ion trap heating, 230°C; scan range, 30–500 m/z. The assay was performed with empty vector as a control.

### Quantitative Real-Time PCR

Total RNA from different tissues (roots and leaves) and different inductive stages was extracted separately using TRIzol reagent (Invitrogen) and pre-treated with RNaseFree DNase (Promega, USA) to eliminate genomic DNA contamination. First-strand cDNA was synthesized using the PrimeScript 1st strand cDNA synthesis kit (TaKaRa Biotechnology, Dalian, China). The real-time quantitative PCR analysis was performed using the SYBR Premix Ex Taq II system (Takara) on an ABI 7500 instrument (Applied Biosystems, Foster City, CA, USA). The primers for qRT-PCR analysis were SQS-qF and SQS-qR (**Table 1**). Actin was used as the endogenous control to normalize expression value. At least three independent experiments were performed for each analysis. The relative amounts of the target genes were evaluated by the relative expression index of mRNA using the 2[^-ΔΔC(T)^] method.

## Results

### Molecular Characterization of the Full-Length *SmSQS2* cDNA

For the cloning of *SmSQS2*, a pair of primer, designed based on the transcriptome sequencing data of *S. miltiorrhiza* Bunge, was used for the amplification of the cDNA of *SmSQS2*. The full-length cDNA of *SmSQS2* was 1 597 bp, with a 1 245 bp ORF, which encodes a 414 amino acid polypeptide, flanked by an 115 bp 5′-untranslated region and a 237 bp 3′-untranslated region. The cDNA sequence was deposited into GenBank with the Accession No.: KM408605. The putative SmSQS2 shared 80.72% similarity with *SmSQS1*, and shared 74.76, 69.47, 78.74, 79.23, 80.24, 81.69, and 80.96% sequence identity with *AtSQS1*, *AtSQS2* (*A. thaliana*), *GgSQS1*, *GgSQS2* (*G. glabra*), *PgSQS1*, *PgSQS2*, *PgSQS3* (*P. ginseng*), respectively.

**FIGURE 1:**
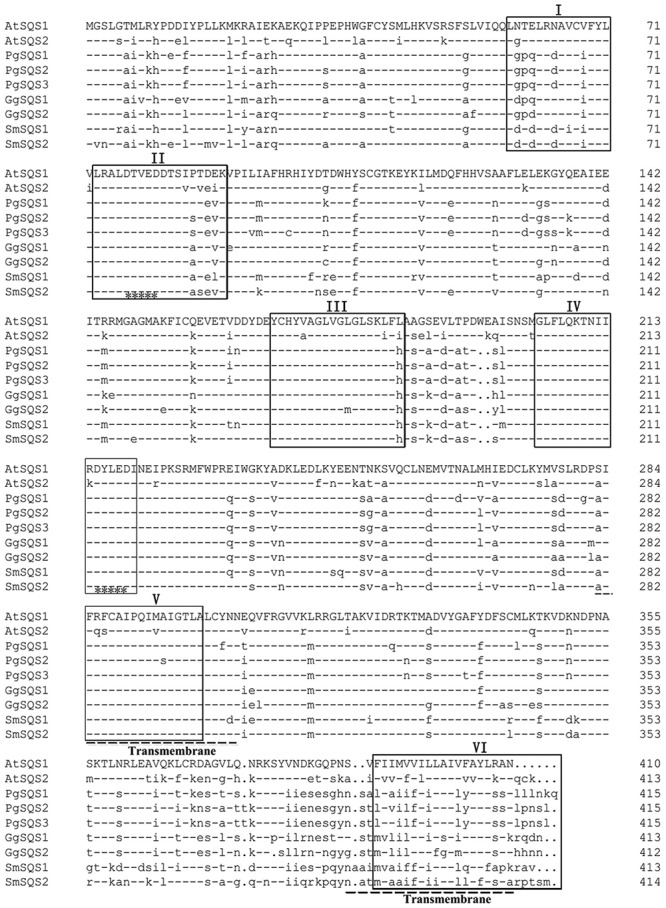
**Amino acid sequence alignment of SmSQS2 and representative squalene synthases (SQSs) from other organisms.** The sequences and their accession numbers are as follows: AtSQS1, *Arabidopsis thaliana*, NP_195190.1; AtSQS2, *A. thaliana*, NP_195191.2; GgSQS1, *Glycyrrhiza glabra*, BAA13083.1; GgSQS2, *G. glabra*, BAA13084.1; PgSQS1, *Panax ginseng*, BAD08242.1; PgSQS2, *P. ginseng*, ACV88718.1; PgSQS3, *P. ginseng*, ACZ71037.1; SmSQS1, *Salvia miltiorrhiza*, ACR57219.1. Squalene synthase conserved domain I, II, III, IV, V, and VI are underlined by “□,” two transmembrane domain are marked out by “—,” and two aspartate-rich regions (DXXXD) that mediate binding of prenyl phosphates are marked out by “^∗^”.

The putative *SmSQS2* protein had a calculated molecular mass of 47.16 kDa and a theoretical isoelectric point of 7.16. Conserved domain database (CDD) search at NCBI indicated the presence of conserved aspartate-rich regions, substrate binding pocket, substrate-Mg^2+^ binding region suggesting that the protein may encode for functional enzyme (**Supplementary Figure S1**). Multiple sequence alignments showed that the putative SmSQS2 had high identity with SQSs from other plants and revealed six highly conserved signature domains (I–VI; **Figure 1**). Domain I is substrate binding pocket or chemical binding site which consists of about 18 amino acids distributed throughout the polypeptide. Domain II is substrate–Mg^2+^ binding site which consists of 10 amino acids divided in two patches of 5 each in which domain V and VI (aspartate rich regions 1 and 2, respectively) are embedded. Domain III is active site, which is composed of about nine amino acid residues. Domain IV is catalytic domain of about 14 amino acids distributed throughout the polypeptide. The catalytic site is composed of the large central cavity formed by antiparallel alpha helices with two aspartate rich regions (DXXXD) on opposite walls (Marrero et al., 1992; Chen et al., 1994; Reipen et al., 1995; Pandit et al., 2000) (**Figure 1**). These residues are considered to play role in binding of prenyl phosphates by binding Mg^2+^ ions.

Squalene synthase proteins are membrane bound enzymes anchored to the endoplasmic reticulum by their highly hydrophobic transmembrane domain located in their C-terminals (Robinson et al., 1993). This region is poorly conserved among SQS proteins. The TMHMMserver 2.0 showed that the putative *SmSQS2* has two transmembrane helices, which were from 281 to 303 and 387 to 409 aa (**Figure 1**; **Supplementary Figure S2**).

**FIGURE 2:**
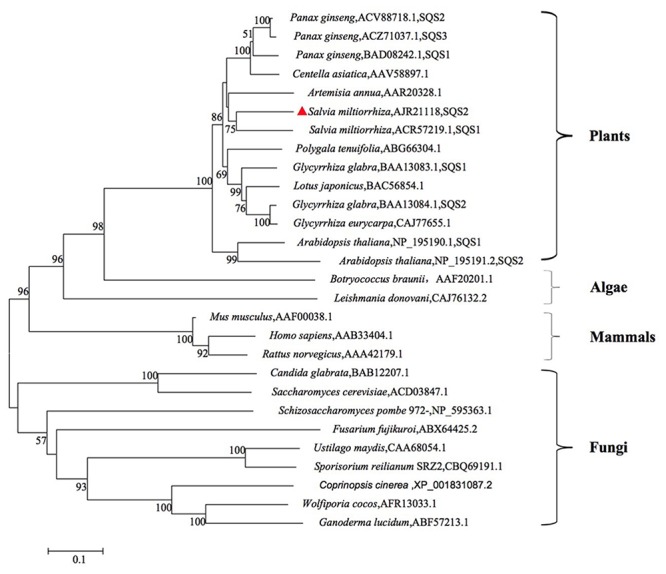
**Phylogenetic tree analysis of SQSs from different organisms constructed by the neighbor-joining method based on 1 000 bootstrap replicates (value for condensed tree ≥ 50 %)**.

The phylogenetic tree was constructed using known SQS sequences from various different types of organisms to investigate the evolutionary relations (**Figure 2**). The phylogenetic tree was divided four main clusters: Plants, Algae, Mammals, Fungi, and SmSQS2 clustered within the Plants. Phylogenetic analysis of putative SmSQS2 showed that it was more closely related to SmSQS1 followed by *Artemisia annua*.

### 3D Structure Analysis

The secondary structure of the putative *SmSQS2* protein was predicted to be mainly α-helix (69.08%), extended strand (7.73%), β-turn (5.80%), and random coil (17.39%). The 3D structure of the putative SmSQS2 was predicted using homology structure modeling on Swiss model server. The sequence used as a template was a human squalene synthase, accession number 3vj9.1. This template showed a sequence identity of 47.59% with the putative SmSQS2 and was the most homologous SQS for which X-ray structure information was available (**Supplementary Figure S3**).

### Characterization of Recombinant Protein

**FIGURE 3:**
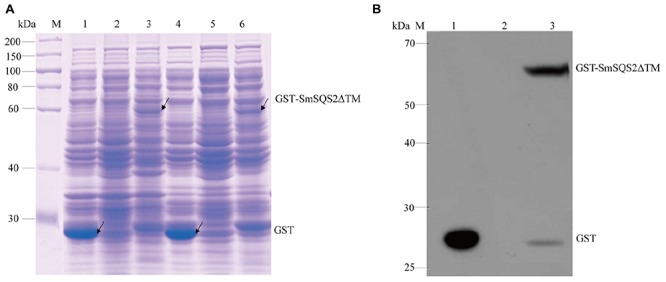
**Characterization of recombinant protein. (A)** SDS-PAGE analysis of recombinant *SmSQS2* protein expressed in *Escherichia coli*. M: protein marker. Lane 1-3( total cell extract), 1: *pGEX-4T-1* bacteria of after induce; 2: *pGEX-4T-1-SmSQS2*Δ*TM* bacteria of before induce;3: *pGEX-4T-1-SmSQS2*Δ*TM* bacteria of after induce; Lanes 4–6(supernatant of the cell lysate treated),4: *pGEX-4T-1* bacteria of after induce; 5: *pGEX-4T-1-SmSQS2*Δ*TM* bacteria of before induce; 6: *pGEX-4T-1-SmSQS2*Δ*TM* bacteria of after induce. **(B)** Western Blot assay of the recombinant *SmSQS2* protein expressed in *E. coli*. M: protein marker; 1: supernatant of the *pGEX-4T-1* ultrasound crushing after induce; 2: supernatant of the *pGEX-4T-1-SmSQS2*Δ*TM* ultrasound crushing before induce; 3: supernatant of the *pGEX-4T-1-SmSQS2*Δ*TM* ultrasound crushing after induce.

Earlier reports suggested that deletion of the hydrophobic C-terminal SQS region facilitated expression of soluble protein (Gupta et al., 2012). To obtain soluble recombinant enzymes, the truncated SmSQS2 in which 28 amino acids were deleted from the carboxy terminus was expressed as GST-Tag named *pGEX-4T-1-SmSQS2*Δ*TM*. The *pGEX-4T-1* vector without the *SmSQS2* and transformed in *E. coli* BL21 (DE3) was used as a control. The recombinant putative protein *pGEX-4T-1-SmSQS2*Δ*TM* was induced with 1 mM IPTG at 30°C for 6 h. After sonication and centrifugation of the bacteria, total protein, and supernatants were separated with 10% SDS-PAGE. SDS-PAGE analysis (**Figure 3A**) showed that the putative protein was successfully expressed in the supernatant and total cell extract, with a molecular mass of approximately 66.46 kDa, while the corresponding protein bands were not found at the expected position (around 66.46 kDa) in the uninduced sample.

Western Blotting showed that the band of recombinant protein (thus including the Gst -tag) was correct. No such band was observed in the control strain. Anti-Gst tag antibody was a ∼66.46 kDa protein band on the membrane (**Figure 3B**). The result of Western Blot analysis indicated that the anti-Gst tag antibody cross reacted with recombinant putative *pGEX-4T-1-SmSQS*Δ*TM* giving a fairly detectable cross reactivity band corresponding to 66.46KDa representing the recombinant fusion protein produced in *E. coli* BL21 (DE3).

### Functional Characterization of the *SmSQS2*

**FIGURE 4:**
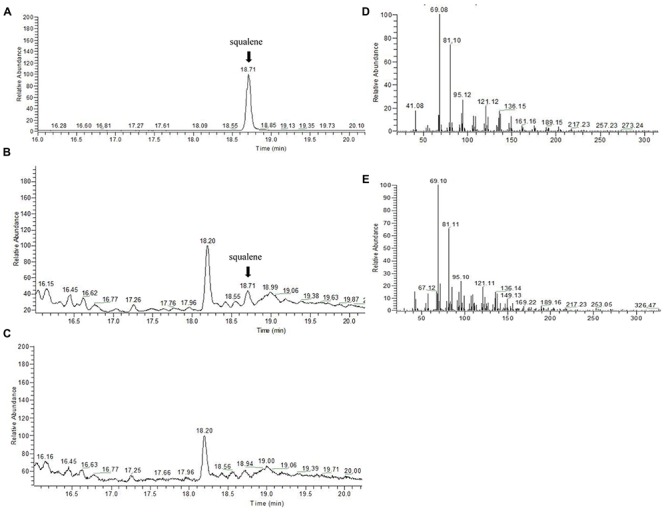
**GC–MS detection of squalene formation catalyzed by recombinant *SmSQS2.* (A) GC-MS analysis of the squalene as standards. (B)**. The reaction products catalyzed by recombinant *SmSQS2*. **(C)** Contol (the empty vecter). **(D)** The mass spectrogram of the squalene. **(E)** The mass spectrogram of the reaction products catalyzed by recombinant *SmSQS2*.

To confirm that the *SmSQS2* gene encoded functional SQS, the activity of the recombinant protein was measured for conversion of FPP to squalene in the presence of NADPH and Mg^2+^. The catalytic product was identified as squalene by GC-MS analysis (**Figure 4**). The TIC peak at 18.71 min in the SmSQS2 chromatogram (**Figure 4B**) corresponds to that at 18.71 min observed in authentic squalene (**Figure 4A**), while no such peak was detected in the vector-control (**Figure 4C**). The detection of major mass fragments of m/z = 69 and m/z = 81 in the 18.71 min peak of SmSQS2 is consistent with those seen in authentic squalene, in contrast to no corresponding fragments observed in the vector-control (**Figures 4D,E**). Thus, these results clearly prove that SmSQS2 codes for squalene synthase.

### Tissue-Specific and Inducible Expression of *SmSQS2*

**FIGURE 5:**
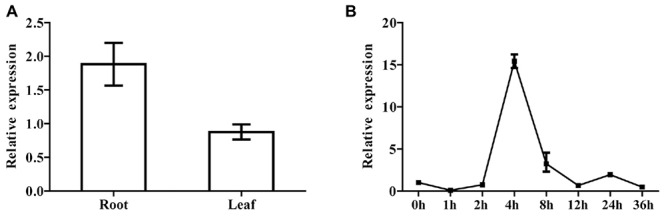
**Tissue-specific and inducible expression of *SmSQS2*. (A)** qRT-PCR analysis of transcript levels of *SQS2* gene in different tissues of *S. miltiorrhiza.* Expression was normalized to that of *Actin*. The expression level of *SmSQS2* in leaf was set to 1. Data are means ± SEM (*n* = 3). **(B)** Expression profile of *SmSQS2* when treated with YE+ Ag^+^ treatment. Expression was normalized to that of *Actin*. The expression level of *SmSQS2* at 0 h was set to 1. Data are means ± SEM (*n* = 3).

For the purpose of studying the expression of SQS gene in each tissue, we examined the *SmSQS2* expression pattern in different tissues using total RNA from leaves and roots. The expression level of leave was set up as a control and the *SmSQS2* expression in other stage was evaluated relative to the leave. Among different tissues of *S. miltiorrhiza* Bunge, the transcript levels of *SmSQS2* were observed in the leaves and roots (**Figure 5A**). There are significant differences between the expression levels for the two tissues by an unpaired *t*-test using GraphPad Prism 6. *SmSQS2* exhibited the higher expression level in the roots than in the leaves (*P* < 0.05; **Figure 5A**).

Moreover, real-time PCR analysis with hairy root at different developmental stages was also performed to examine the changes in the expression of *SmSQS2* genes upon biotic (yeast extract) and abiotic (Ag^+^) treatment. Specifically, YE+ Ag^+^ caused a significant increase in *SmSQS2* levels in *S. miltiorrhiza* hairy-root. The expression level of 0 h was set up as a control and the *SmSQS2* mRNA expression in other stage was evaluated relative to the 0 h. Interestingly, the dynamic range of the induction varied. The levels of *SmSQS2* expression increased at first, peaked at 4 h, then decreased gradually (**Figure 5B**). It is indicated that YE+ Ag^+^ can induced the expression of *SmSQS2*.

## Discussion

*Salvia miltiorrhiza* Bunge, one of the plants of the genus Salvia (Labiatae), has been used primarily in traditional Chinese medicine herb for the treatment of cardiovascular diseases. The main effective elements of Radix *S. miltiorrhizais* Bunge are Tanshinones and Phenolic acids. In addition, the bioactive compounds of *S. miltiorrhizais* Bunge including sterols, which are essential for all eukaryotic organisms to function (Bloch, 1992), and triterpenoids which seemed to be potentially promising as an antiatherogenic agent, especially when more pharmacological, toxicological work is conducted (Liu et al., 2011). Because of their important medicinal activity, most of the Danshen researches have been focused on the sterol and triterpenoid biosynthesis and the identification of some specific enzymes with sterol and triterpenoid biosynthesis from the MVA pathway. Squalene synthase catalyzes the first enzymatic step in sterol and triterpenoid biosynthesis from the central isoprenoid pathway. SQSs were studied as a key enzyme for the biosynthesis of squalene as an intermediate for the production of sterols, hopanoids or other triterpenoids.

To gain new insights into the role of SQS in the isoprenoid biosynthetic pathway in higher plants, we isolated cDNA of *SmSQS2* was 2 529 bp containing an ORF of 1 597 bp encoding a protein consisting of 414 amino acids. The predicted molecular weight of deduced polypeptide of SmSQS2 was 47.16 kDa. SQS is generally accepted to be a monomeric enzyme with molecular weight in a range from 40 to 50 kDa. However, some exceptions are indeed found only in prokaryotes. The molecular weight of *Staphylococcus aureus* SQS is 56 kDa (Holden et al., 2004) while SQS from *Botryococcus braunii* is 7.3 kDa (Kaneda et al., 2001).

Alignment of SmSQS2 with other SQSs and comparison with different sources of SQSs showed that six highly conserved regions were present in the SmSQS2 (**Figure 1**). These consensus regions are predicted or even have been proven to be important for the SQS activity based on the kinetic studies with site-directed mutagenesis or the analysis of a crystal structure of human SQS (Gu et al., 1998; Pandit et al., 2000). Interestingly, *SmSQS2* not only have a membrane-spanning helix within the C-terminal hydrophobic sequences, but also have additional membrane-spanning helices near domain V. This second transmembrane domain was also reported in SQS from closely related sequences of *G. glabra*, *M. truncatula*, *L. japonicas*, *E. tirucalli*, and *P. ginseng* (Kim et al., 2011). In contrast, *Arabidopsis* (Busquets et al., 2008) and mammalian SQS proteins do not have this additional trans-membrane domain.

The SQS C-terminal region is hydrophobic and may function as an anchor in the endoplasmic reticulum membrane. Low expression levels of SQS polypeptide were observed in *E. coli* cells that contained the putative full-length *SmSQS* cDNA (data not shown). On the other hand, due to truncated C-terminus, measurable levels of SQS polypeptide were observed in the extracts of *E. coli* that expressed the truncated *SmSQS* (*PGEX-4T-SmSQS2*Δ*TM*). This resulted in the formation of a new polypeptide with an expected molecular mass of 66.46 kDa (including 24 kDa Gst Tag) on the SDS-PAGE gel (**Figure 3A**). GC–MS analysis confirmed that the recombinant SQS proteins could catalyze the formation of squalene from FPP and NADPH is essential requirement of the reaction (**Figure 4**). Somewhat similar results also reported in yeast SQS (LoGrasso et al., 1993), tobacco SQS (Hanley et al., 1996; Devarenne et al., 2002), capsicum SQS (Lee et al., 2002), *W. somnifera* SQS (Gupta et al., 2012) and *Selaginella moellendorffii* SQS (Jiang et al., 2015).

Results of qRT-PCR analyses of *SmSQS2* expression demonstrated that *SmSQS2* has tissue specific expression with higher expression in roots than in leaves (**Figure 5A**). The similar results were found with the expression of some other plants of the *Taxus cuspidata SQS* genes (Huang et al., 2007), while some different observation were seen in *W. somnifera* where *SQS* has tissue specific expression with highest expression in leaves and lowest in roots (Gupta et al., 2012). Therefore, the *SQS* expression patterns may be different during the plants growth period.

It is reported that tanshinone biosynthesis-related genes expression were regulated and tanshinones content was also enhanced after treated with YE+ Ag^+^ (Ge and Wu, 2005). The combined treatment of UV-B irradiation and MeJA exhibited synergistic effects on the expression levels of *3-hydroxy-3-methylglutaryl-CoA reductase* (*SmHMGR*) involved in MVA pathway and *geranylgeranyl diphosphate synthase* (*SmGGPPS*) genes in the tanshinone biosynthetic pathway (Wang et al., 2016). All MVA pathway related genes from *S. miltiorrhiza* exhibited a significant increase in expression levels at 12 hpi, but this was followed by a significant drop at 24 hpi, and a return to expression levels only slightly higher than the control (0 hpi) at 36 hpi, representing a rapid but transient response to elicitation (YE+ Ag^+^; Gao et al., 2014). These reported indicated that proper elicitors might induce multiple enzymes in the secondary metabolites biosynthesis pathway. Therefore, we investigated the expression of SQS genes under combined YE+ Ag^+^ treatments. Real-time PCR analysis with *S. miltiorrhiza* Bunge hairy root was up-regulated in the expression of *SmSQS2* genes upon biotic (yeast extract) and abiotic (Ag^+^) treatment peaked at 4 h, and was 15-hold higher than control group (**Figure 5B**). YE+ Ag^+^ caused a significant increase in *SmSQS2* levels in *S. miltiorrhiza* Bunge hairy root. These data, thus, provide an additional evidence that SQS2 may exhibit an important role in sterol and triterpenoid synthesis.

In summary, due to the functions of SQS in the sterol and terpenoid biosynthesis in *S. miltiorrhiza* Bunge, we report the isolation of the full-length cDNA encoding *SmSQS2* and the functional analysis of the recombinant SmSQS2 protein. It will not only be beneficial for better understanding of this pathway but will also provide molecular wealth for biotechnological improvement of this medicinal plant.

## Author Contributions

QR and DJ participated in the design of the study, data analysis, and prepared the manuscript. YC and LZ conducted experiments. YS, QY, HL, and YZ participated in the design of the study and LH is responsible for the overall supervision of the work. All authors read and approve the final manuscript.

## Conflict of Interest Statement

The authors declare that the research was conducted in the absence of any commercial or financial relationships that could be construed as a potential conflict of interest.

## Footnotes

1 http://www.ncbi.nlm.nih.gov/

2 http://www.ncbi.nlm.nih.gov/gorf/gorf.html

3 http://ca.expasy.org/tools

4 http://www.cbs.dtu.dk/services/TMHMM-2.0/

5 http://swissmodel.expasy.org/

## Supplementary material

FIGURE S1 **Conserved domains of *SmSQS2* protein as determined using NCBI conserved domain database indicating the presence of conserved isoprenoid domain and other substrate binding domains**.

FIGURE S2 **The predicted transmembrane regions of SmSQS2**.

FIGURE S3 **The predicted 3D structure model of SmSQS2**.
