# Supplementary figures and images for: Molecular Cloning and Functional Analysis of Squalene Synthase 2(SQS2) in Salvia miltiorrhiza Bunge
Source: Front Plant Sci. 2016 Aug 24;7:1274. doi: 10.3389/fpls.2016.01274 (PMC4996051; doi:10.3389/fpls.2016.01274)

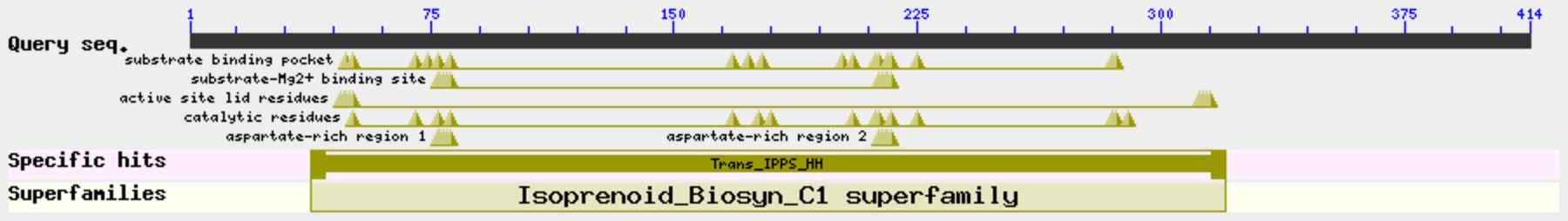

Supplement: FIGURE S1 — Conserved domains of SmSQS2 protein as determined using NCBI conserved domain database indicating the presence of conserved isoprenoid domain and other substrate binding domains. [file Image_1.JPEG]

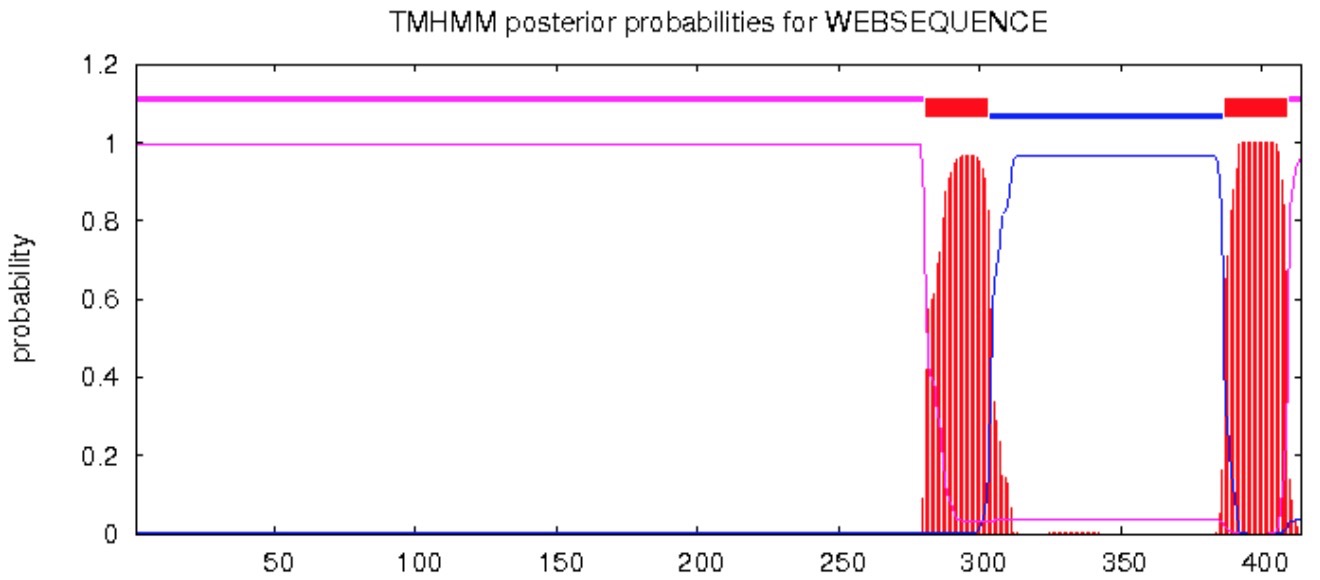

Supplement: FIGURE S2 — The predicted transmembrane regions of SmSQS2. [file Image_2.JPEG]

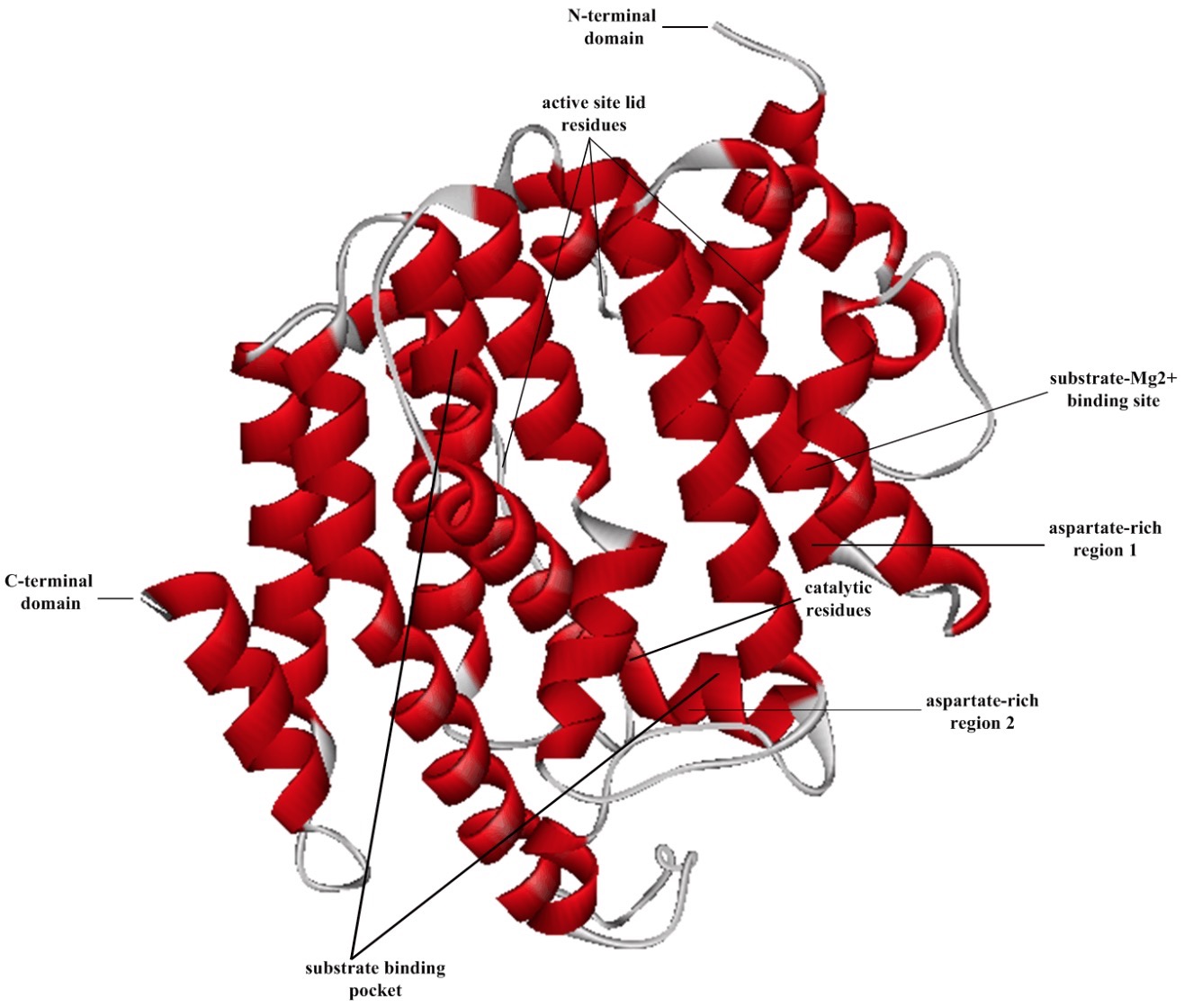

Supplement: FIGURE S3 — The predicted 3D structure model of SmSQS2. [file Image_3.JPEG]
